# MARK4 and MARK3 associate with early tau phosphorylation in Alzheimer’s disease granulovacuolar degeneration bodies

**DOI:** 10.1186/2051-5960-2-22

**Published:** 2014-02-17

**Authors:** Harald Lund, Elin Gustafsson, Anne Svensson, Maria Nilsson, Margareta Berg, Dan Sunnemark, Gabriel von Euler

**Affiliations:** Department of Neuroscience, iMed, CNS and Pain Södertälje, AstraZeneca Research and Development, Södertälje, Sweden; Department of Clinical Neuroscience, Center for Molecular Medicine, Karolinska Institutet, Stockholm, 17176 Sweden

**Keywords:** Alzheimer’s disease, MARK, Phosphorylation, Granulovacuolar degeneration bodies, Tau

## Abstract

**Background:**

The progression of Alzheimer’s disease (AD) is associated with an increase of phosphorylated tau in the brain. One of the earliest phosphorylated sites on tau is Ser^262^ that is preferentially phosphorylated by microtubule affinity regulating kinase (MARK), of which four isoforms exist. Herein we investigated the expression of MARK1-4 in the hippocampus of non-demented elderly (NDE) and AD cases.

**Results:**

*In situ* hybridization revealed a uniform, neuronal distribution of all four isoform mRNAs in NDE and AD. Immunohistochemical analyses using isoform-selective antibodies demonstrated that MARK4 in a phosphorylated form colocalizes with p-tau Ser^262^ in granulovacuolar degeneration bodies (GVDs) that progressively accumulate in AD. In contrast MARK4 is largely absent in the neuronal cytoplasm. MARK3 was localized to a subset of the GVD-containing neurons and also had a weak general cytoplasmic neuronal staining in both NDE and AD. These results suggest that in AD, phosphorylated MARK3 and MARK4 are sequestered and proteolysed in GVDs. MARK1 and MARK2 were absent in GVDs and exhibited relatively uniform neuronal expressions with no apparent differences between NDE and AD.

**Conclusion:**

We found that the phosphorylated and fragmented forms of MARK4 and to some extent MARK3 are present in GVDs in AD, and that this expression is highly correlated with phosphorylation of tau at Ser^262^. This may represent a cellular defense mechanism to remove activated MARK and p-tau Ser^262^ from the cytosol, thereby reducing the phosphorylating effect on tau Ser^262^ that appears to be a critical step for subsequent neurodegeneration.

## Background

Tau protein was identified as the principal component of Alzheimer’s disease (AD) neurofibrillary tangles (NFTs) in the 1980s [1–3]. This finding led to the identification of many kinases that have the ability to phosphorylate tau and the description of more than 45 phosphorylation sites present on paired helical filament (PHF) tau [4–6]. Indeed, phosphorylation at specific tau epitopes is critical for neurofibrillary tangle formation and subsequent neurodegeneration [7, 8]. One of the earliest sites on tau to become phosphorylated in the AD brain is Ser^262^[9], which is located in the KXGS motif in one of the microtubule repeat domains, and that can be phosphorylated by MARK1-4 [10–13], a family of four highly conserved kinases [10, 12, 14].

It is known from *in vitro* studies that MARK phosphorylation of tau at the Ser^262^ site causes detachment of tau from microtubules and their subsequent destabilization makes tau available for further phosphorylation by other kinases [10, 15], and MARK phosphorylation can induce mis-sorting of phosphorylated tau [16]. Tau Ser^262^ phosphorylation and mislocalization are early events in a mouse model of tau pathology [17], and studies in *Drosophila* have demonstrated a crucial role of the MARK phosphorylation site on tau for neurodegeneration [18, 19].

A previous study that examined MARK expression in the human brain reported increased MARK1 expression in AD, but lacked a robust confirmation of the isoform-specificity of the antibody used [20]. We recently succeeded in developing and identifying specific antibodies towards each of the four MARK isoforms. Using these specific antibodies and a monoclonal antibody towards unphosphorylated tau we were able to demonstrate an increased interaction of MARK2 and MARK4 in AD hippocampal tissue compared to controls using the *in situ* proximity ligation assay [13, 21].

Granulovacuolar degeneration bodies (GVDs) are double membrane vacuoles present in neurons, having an immunohistochemical signature that suggests that they derive from the autophagic system [22]. GVDs also stain for cytoskeletal proteins such as neurofilament, tubulin tau and tau kinases [1, 23–28]. GVDs have been shown to be more frequent in AD brains compared to in age-matched controls [29], and a recent study suggests that GVD accumulation is specific to AD, since GVD frequency correlated with every measure of AD severity but was not different in any other non-AD tauopathies compared to control brains [30].

In the present study we characterized the intracellular localization of the four MARK isoforms and investigated whether their expression levels were elevated in the hippocampus in AD. We observed abundant neuronal mRNA expression of all MARK isoforms in both AD and NDE cases. At the protein level we determined that MARK1 and MARK2 were abundantly expressed in neuronal cytoplasm, but that expression levels did not increase in AD. In addition to a general cytoplasmic expression that did not change in AD, MARK3 was detected in a minor fraction of GVDs that are evident in neurons in AD. The expression of MARK4 was below the detection level in normal brain tissue, but was highly present in a phosphorylated form in GVDs in AD, where it colocalized with tau Ser^262^ phosphorylation.

## Methods

### Human brain tissues

All studies of human tissue have been reviewed and approved by the ethical review board in Stockholm, Sweden. All human brain tissues included in this study were acquired from the Netherlands Brain Bank where informed consent for donated tissue had been given by all patients or their next of kin. Neuropathological diagnosis was based on NIA-Reagen criteria with both CERAD and Braak staging. Case and tissue details are summarized in Table 1. Both paraffin embedded (4 μm sections) and fresh frozen tissue (8–10 μm sections) were used.

**Table 1:** **Case characteristics**

Case #	Brain region	Format	Gender	Age	Diagnosis	Braak stage	PMI (h:m)	pH CSF	Brain weight (g)	ApoE genotype
***In situ*** **hybridization**										
6	HC	FF	M	85	NDE	I	4:15	6.68	1181	4:4
7	HC	FF	F	91	NDE	I	7:45	6.90	1074	3:3
27	HC	FF	F	84	AD	V	5:55	6.42	1217	3:3
29	HC	FF	F	89	AD	V	4:40	6.28	1022	4:3
19	HC	FF	F	82	AD	VI	4:00	6.66	1110	4:4
21	HC	FF	F	89	AD	VI	4:30	6.35	1185	4:3
**Immunohistochemistry/Immunofluorescence**										
1	HC	FF	M	74	NDE	0	8:00	6.75	1317	3:2
22	HC	FF	F	77	NDE	I	5:30	6.74	1343	4:3
4	HC	FF	M	78	NDE	I	6:55	6.42	1332	3:3
30	HC	FFPE	F	83	NDE	I	5:30	6.48	1294	3:2
6	HC	FFPE	M	85	NDE	I	4:15	6.68	1181	4:4
7	HC	FFPE, FF	F	91	NDE	I	7:45	6.90	1074	3:3
9	HC	FFPE	M	81	NDE	II	5:30	6.46	1348	3:3
25	HC	FFPE, FF	M	87	NDE	II	4:55	6.31	1052	3:3
10	HC	FFPE	M	82	NDE	IV	10:00	6.53	1528	4:3
38	HC	FF	F	95	AD/LBV	IV	5:25	6.10	1091	3:3
13	HC	FFPE, FF	M	64	AD	IV	6:00	6.62	1128	3:3
26	HC	FF	F	86	AD	IV	5:05	6.62	998	4:3
31	HC	FFPE	F	94	AD	IV	5:05	6.52	1170	4:3
11	HC	FF	F	71	AD	V	5:30	6.36	1125	4:3
24	HC	FF	M	75	AD	V	5:15	6.39	1178	4:3
32	HC	FF	F	78	AD	V	4:50	6.22	1105	4:4
33	HC	FFPE	F	82	AD	V	4:35	6.49	1104	4:3
14	HC	FFPE	F	84	AD	V	7:15	6.58	1129	4:3
12	HC	FF	F	84	AD	V	4:50	6.67	1092	4:4
28	HC	FFPE	M	87	AD	V	6:10	6.14	1088	3:3
16	HC	FF	F	88	AD	V	5:10	6.62	1075	4:3
29	HC	FFPE	F	89	AD	V	4:40	6.28	1022	4:3
34	HC	FFPE	F	67	AD	VI	5:50	6.75	945	3:3
18	HC	FFPE, FF	F	68	AD	VI	3:50	6.50	1095	3:2
35	HC	FFPE	F	69	AD	VI	4:45	6.33	862	3:3
37	HC	FF	F	87	AD	VI	4:00	6.80	1048	4:3
21	HC	FF	F	89	AD	VI	4:30	6.35	1185	4:3
**Western blot**										
1	HC	FF	M	74	NDE	0	8:00	6.75	1317	3:2
4	HC	FF	M	78	NDE	I	6:55	6.42	1332	3:3
6	HC	FF	M	85	NDE	I	4:15	6.68	1181	4:4
7	HC	FF	F	91	NDE	I	7:45	6.90	1074	3:3
27	HC	FF	F	84	AD	V	5:55	6.42	1217	3:3
29	HC	FF	F	89	AD	V	4:40	6.28	1022	4:3
19	HC	FF	F	82	AD	VI	4:00	6.66	1110	4:4

AD = Alzheimer’s disease; FF = fresh frozen; FFPE = formalin-fixed paraffin embedded; HC = hippocampus; LBV = Lewy body variant; NDE = non-demented elderly; PMI = post mortem interval; CSF = cerebrospinal fluid.

### *In situ* hybridization

*In situ* hybridization was performed on 2 NDE and 4 AD cases. ^35^S-UTP labeled cRNA probes were synthesized by *in vitro* transcription with the MAXIscript Kit (Ambion) from a synthetic DNA fragment corresponding to part of the coding sequence of human MARK1 (nucleotides 1537–2116 of accession no NM_018650), human MARK2 (1629–2228 of NM_001039469), human MARK3 (1823–2412 of NM_001128918) or human MARK4 (1181–1781 of NM_031417) cloned into a pGEM-5Z (+) vector (GeneART). Probes were designed to minimize cross-reactivity towards the other isoforms as summarized in Table 2. Probes were synthesized in both antisense and sense directions and hybridized to adjacent sections to control for labeling specificity. The rest of the protocol was conducted as previously described [31]. Briefly, sections were fixed with 4% paraformaldehyde (PFA), rinsed 3 times in 2× standard sodium citrate buffer (2× SSC), equilibrated in 0.1 M triethanolamine, and treated with 0.25% acetic anhydride in 0.1 M triethanolamine. Sections were equilibrated in chloroform and dehydrated through an ethanol series. Hybridization with [^35^S]-labeled cRNA probes was performed at 59°C overnight under a coverslip. Following hybridization sections were treated with 20 μg/ml RNase A for 45 minutes at 37°C, washed in a series of decreasing SSC-containing solutions with a final high stringency wash of 0.1× SSC and 1 mM DTT at 69°C. Sections were then dehydrated and exposed to Kodak Biomax MR-2 film, dipped in NTB2 emulsion (Kodak) and exposed at 4°C prior to development and counterstaining with hematoxylin.

**Table 2:** **Cross-reactivity of probes used for** ***in situ*** **hybridization**

	MARK1	MARK2	MARK3	MARK4
Length	580 bp	600 bp	590 bp	601 bp
MARK1	100%	38.7%	55.6%	50.4%
MARK2	51.9%	100%	36.1%	52.6%
MARK3	55.9%	39.3%	100%	52.2%
MARK4	48.1%	42.3%	46.6%	100%

Percentages are overall sequence identity of probes toward each MARK family isoform.

### Primary antibodies

The following rabbit antibodies were used: human MARK1 (AGG6175; AstraZeneca), raised against the peptide DGSEAYRPGT; human MARK2 (AGG6218; AstraZeneca), raised against SVLSTSTNRSRNS; human MARK3 (#9311; Cell Signaling); human MARK4 (#4834; Cell Signaling); phospho-MARK family (PA5-17495; Pierce); p-tau Ser^262^ (AGG5759; AstraZeneca), raised against KSKIGS*TENLKHQPGGC (*phosphorylated) and affinity-purified first against the phosphorylated peptide and then against corresponding non-phosphorylated peptide; p-tau Ser^422^ (EPR2866; Epitomics); CK1δ (ab37971; AbCam). In addition we used the following mouse monoclonal antibodies: p-tau Ser^202^/Thr^205^ (AT8; Innogenetics), p-tau Ser^212^/Thr^214^ (AT100, Innogenetics), total-tau (tau-13; SantaCruz) and β-actin (AC-15; Sigma).

### Immunohistochemistry

Immunohistochemistry was conducted as previously described [31]. Briefly, stainings were performed using an Ventana Discovery XT automated staining module using the OmniMap DAB kit (Ventana Medical Systems) according to the manufacturer’s instructions. Sections were scanned with a NanoZoomer 2.0-HT slide scanner (Hamamatsu) and images were captured from the digital sections using NDP.view software.

### Immunofluorescence

Multi-label immunofluorescent staining with two or three rabbit antibodies was carried out using the tyramide signal amplification (TSA) kit (PerkinElmer) according to the manufacturers’ instructions. This allows for a very extensive dilution of the primary antibody, reducing cross-reactivity of the second secondary antibody to the first primary antibody to a minimum. Double-labeling was carried out with one TSA-reaction followed by normal immunofluorescent staining. Triple-labeling entailed two TSA-reactions followed by normal immunofluorescent staining. Secondary antibodies for TSA-reactions were swine anti-rabbit- HRP (Dako). For immunofluorescence donkey anti-rabbit conjugated to FITC or Cy3 (Jackson ImmunoResearch) or goat anti-rabbit Alexa Fluor 594 (Invitrogen) were used. Images were captured using a Leica DMI6000 CS confocal microscope connected to a Leica TCS SP5 scanner.

### Quantification of colocalization

Colocalization of MARK3 or MARK4 and GVD-marker CK1δ was evaluated using ImageJ 1.47v software. Briefly, Z-stack images of individual neurons were captured using confocal microscopy (3–12 neurons/case; 4–6 cases), thresholded and converted to 8-bit images. Green and red channel images were then superimposed on each other and colocalization determined using the Green and Red Puncta Colocalization Macro (Developed by D. J. Shiwarski, R. K. Dagda and C.T. Chu). The number and size of single-stained and double-stained particles were then determined using the Particle Analyzer tool in ImageJ.

### Cell culture and transfections

The human embryonic kidney cell line HEK293 was purchased from ATCC (CRL-1573). Cells were grown in DMEM/F12 medium with Glutamax (Invitrogen) and supplemented with 10% heat-inactivated fetal calf serum (FCS, Hyclone). Cells were seeded in T225 flasks (Corning) and transfected with a total of 63 μg plasmid cDNA (HA-tagged MARK3 or MARK4)/flask at 70% confluence. Lipofectamine LTX with PLUS reagent (Invitrogen) was used for transfections according to the manufacturer’s manual. Briefly, PLUS reagent was mixed with plasmid DNA in cell culture medium without serum and incubated for 5 min. Lipofectamine LTX was added and incubated an additional hour. The complexes were added to the cells and transfections were performed for 24 hours.

### Western blotting

The transfected HEK293 cells were lysed in buffer containing 50 mM TrisHCl, pH 7.2, 150 mM NaCl, 1 mM EDTA, 1% Triton X-100, 10 mM NaF, 1 mM Na_3_O_4_V, and 1 complete protease inhibitor cocktail tablet (Roche)/10 ml buffer. The cells were incubated with lysis buffer for 5 min on ice before scraping from the wells. Samples were incubated at least overnight at −80°C before being thawed on ice and centrifuged at 14000 rpm at 4°C.

100–150 mg of human fresh frozen samples (sectioned adjacently to samples used for *in situ* hybridization and immunohistochemistry) were lysed on ice in ice-cold lysis buffer containing 50 mM Tris acetate, pH 7.4, 5 mM EDTA, 5 mM EGTA and one complete protease inhibitor cocktail tablet (Roche)/10 ml buffer and vortexed. Tissue extraction reagent (Invitrogen) with one complete protease inhibitor cocktail tablet/10 ml was added before sonicating the samples using a Sonifer cell disrupter B15 (Branson) in 5 s intervals placing the samples on ice in between pulsing. Samples were centrifuged at 1000 × g at 4°C for 5 min. The protein contents in supernatants from cell lysates and human tissue extractions were measured using the BCA Protein Assay kit (Pierce). 1 μg proteins from cell lysates and 18 μg proteins from human tissue extractions, respectively, were separated in NuPAGE Novex 4-12% Bis-Tris mini gels (Invitrogen) and transferred to PVDF membranes using the iBlot dry blotting device (Invitrogen). Membranes were blocked in Starting Block T20 PBS Blocking Buffer (Thermo Scientific) before being incubated overnight with primary antibodies at 4°C in PBS containing 0.05% Tween20 (PBS-T). Membranes were washed with PBS-T and incubated with horseradish-peroxidase (HRP)-conjugated secondary antibodies (Amersham Biosciences). After washing, the membranes were developed using the Amersham enhanced chemiluminescence (ECL) western blotting detection system (GE Healthcare). Average den-sities of the bands above background levels were measured using a BioRad GS-800 Calibrated Densitometer using Quantity One 4.6.4 software. The optical densities for MARK3 and MARK4 were normalized to β-actin.

## Results

### Widespread neuronal expression of MARK1-4 mRNA in hippocampus

**Figure 1:**
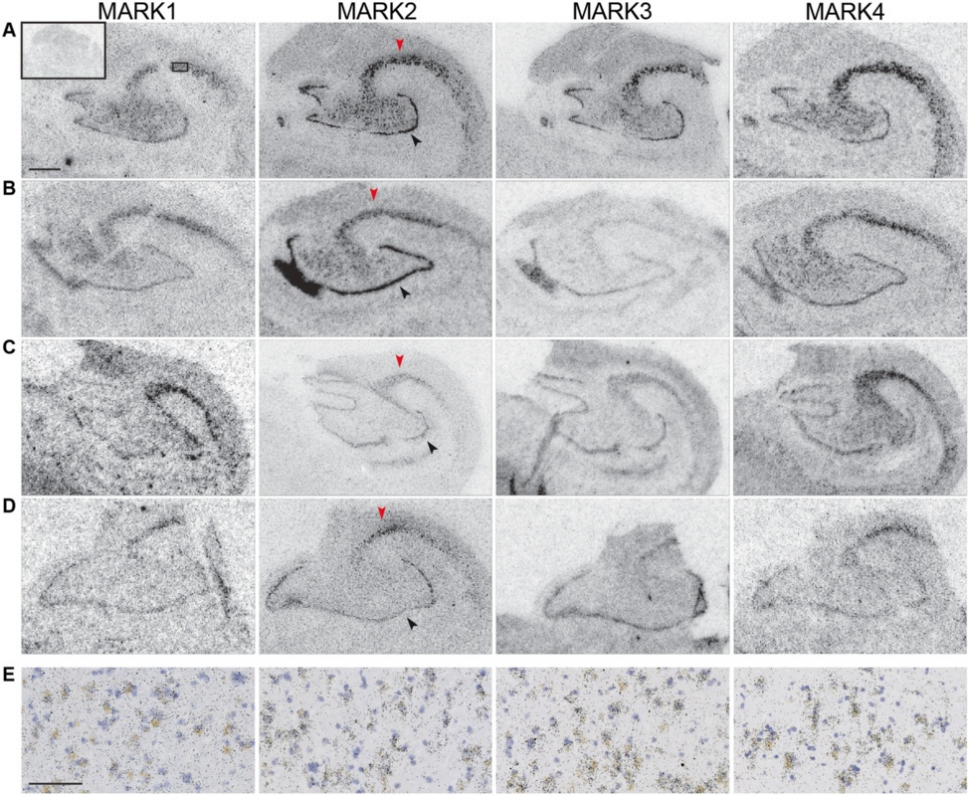
**Characterization of MARK 1–4 mRNA expression by** ***in situ*** **hybridization in the hippocampus.** *In situ* hybridization towards each individual isoform of MARK in fresh-frozen sections of hippocampus from two representative NDE cases (**A**, #6 and **B**, #7) and two representative AD cases (**C**, #27 and **D**, #29), revealing expression and strong overlap of all four isoforms in the dentate gyrus (black arrow head) and all CA fields. The CA2 field is denoted with red arrowheads in the MARK2 panel. Sections hybridized with sense-transcribed probes were used as negative control, with a representative picture presented in the inset. **(E)** Photomicrograph of emulsion-dipped sections counterstained with hematoxylin from the case in **A**, from the area indicated by the box in **A**. Scale bars: **(A)** 1 mm; **(E)** 100 μm.

To study mRNA expression of MARK1-4 in the hippocampus we performed *in situ* hybridization on sections from NDE and AD cases with specific probes towards each individual MARK isoform (Figure 1A-D). Hybridization was particularly strong in neuronal layers and was intense in the dentate gyrus and cornu ammonis (CA), being weaker in the subiculum and entorhinal cortex. Emulsion-dipped and counter-stained sections confirmed that expression was localized to neurons (Figure 1E). Strikingly, the mRNA expression pattern was practically identical between isoforms and there was similar expression in both NDE and AD cases.

### MARK3 and MARK4 are elevated in AD

**Figure 2:**
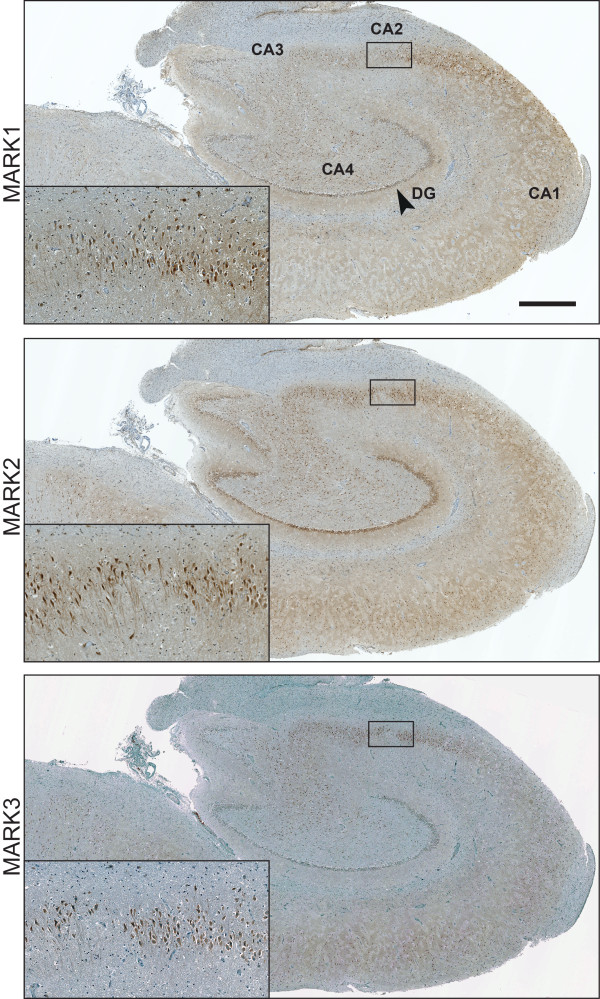
**Expression of MARK1-3 in hippocampal brain sections.** Low magnification micrographs of three adjacent hippocampal sections from an NDE case (#7), with antibodies towards MARK1-3. MARK1 expression is particularly strong in the CA2-layer, with apical dendrites and cell bodies intensely labeled in this area. Less expression is detectable in the CA3, CA4 and DG. MARK2 expression is more homogenously expressed in the CA layers of the hippocampus, particularly strong in CA2 and dentate gyrus (DG), although considerable inter-individual difference existed for this isoform. MARK3 expression on the cytoplasmic level is only detectable in the CA2 layer. Inset images are boxed areas in the CA2 layer. High magnification images are included in Figure 2. Scale bar: 1 mm.

**Figure 3:**
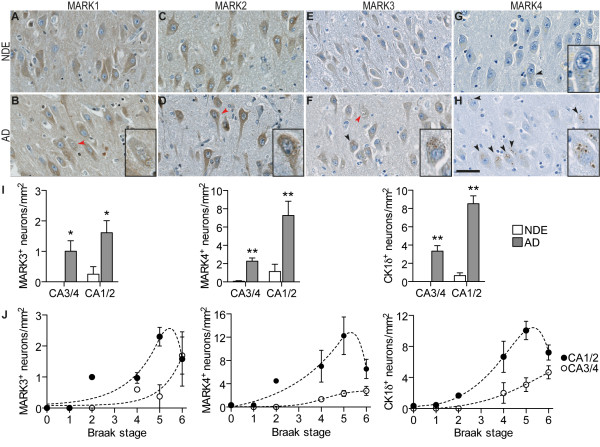
**Immunohistochemical characterization of MARK1-4 protein expression in the hippocampus. (A-H)** Photomicrographs of immunohistochemical staining from the CA2 area of representative NDE and AD cases (#9, #7, #13, #18 and #34) with specific antibodies for each MARK isoform. Neurons containing granulovacuolar degeneration bodies (GVDs) are marked with arrowheads and magnified in insets. MARK1 and MARK2 antibodies reveal a diffuse cytoplasmic staining but no positive staining in GVDs (red arrowheads). Black arrowheads indicate positive staining in GVDs as this is observed in all GVDs with the MARK4 antibody and in some but not all GVDs with the MARK3 antibody. Scale bar: 50 μm. **(I)** Quantification of the number of MARK3, MARK4 or CK1δ neurons with granular immunoreactivity in the CA1-CA2 area or CA3-CA4 area. n = 4–5 NDE; 7–10 AD. Data depicts means ± SEM. *p < 0.05, **p < 0.01 by Student’s unpaired t-test. **(J)** Data in **(I)** plotted against Braak stage. n = 1(Br0); 2-3(Br1); 1(Br2); 2(Br4); 2-5(Br5); 3(Br6).

To assess the protein expression of individual MARK isoforms we used a set of isoform- specific antibodies that was verified in a previous study [13] to stain hippocampus sections of several NDE and AD cases (Table 1). The general localization of MARK1-3 in the hippocampus is depicted in Figure 2. MARK1 and MARK2 immunoreactivity was recorded in the cytoplasm of CA neurons and in the neuropil (Figure 3A-D), the staining intensity being similar in NDE and AD cases. MARK3 immunoreactivity was weak in the cytoplasm but in some cases stained granular deposits in certain neurons (Figure 3E-F). These deposits were observed in all AD cases, particularly in the CA1-CA2 region and more rarely in the CA3-CA4 region. Only a few stained deposits were observed in NDE cases and then only in CA1-CA2. The MARK4 antibody produced no detectable diffuse immunoreactivity in the cytoplasm of either NDE or AD cases, but stained granular deposits in neurons that were particularly numerous in CA1-CA2 and to a lesser extent in CA3-CA4. These MARK4-positive granules were particularly abundant in AD cases and were practically absent in NDE cases (Figure 3G-H).

To quantify expression levels we counted the number of neurons that contained MARK3 or MARK4 immunoreactive granules in the CA1-CA2 and CA3-CA4 areas. Since the granules resembled granulovacuolar degeneration bodies (GVDs) [30, 32], we stained sections in parallel using the GVD-marker CK1δ [28] and counted the number of immunoreactive neurons (Figure 3I). We found a remarkable enhancement in all three markers in AD cases compared to NDE cases in both CA1-CA2 and CA3-CA4. The number of MARK4 and CK1δ neurons were very similar in all areas whereas MARK3 immunoreactivity was only found in 20% of the number of MARK4^+^ or CK1δ^+^ neurons. Interestingly, the number of MARK3^+^, MARK4^+^ or CK1δ^+^ neurons appeared to be Braak-stage dependent. The number of immunoreactive neurons increased with all markers from Braak stage 0-V after which a small decrease between Braak stage V and VI was observed (Figure 3J). GVDs were not stained with either MARK1 or MARK2 antibodies (Figure 3B, D).

**Figure 4:**
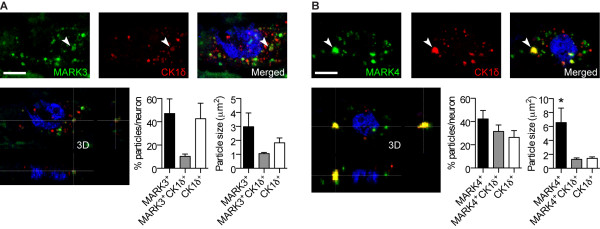
**Colocalization analysis of MARK3 and MARK4 with GVD-marker CK1δ.** Double-immunofluorescence with **(A)** MARK3 (FITC) or **(B)** MARK4 (FITC) and GVD-marker CK1δ (Alexa Fluor 594, red) in GVD-containing neurons in the CA1-CA2 area. Images are compressions of a Z-stack (first three images) and a 3D-reconstruction of a Z-stack imaged by confocal microscopy showing colocalization in the GVD indicated by arrowheads. Bar graphs show the percentage of intracellular granules that were single stained with MARK3 (MARK3^+^), MARK4 (MARK4^+^) or CK1δ (CK1δ^+^), or double-stained with MARK3 and CK1δ (MARK3^+^CK1δ^+^) or MARK4 and CK1δ (MARK4^+^ CK1δ^+^), as well as the average size of these granules. MARK4^+^ granules were significantly larger than MARK4^+^CK1δ^+^ and CK1δ^+^ granules. MARK3^+^, MARK3^+^CK1δ^+^ or CK1δ^+^ granules size was not significantly different. Data depict means ± SEM. *p < 0.05, by 1-way ANOVA with Bonferroni’s Multiple Comparison test, (n = 4–6 cases). Scale bars: 10 μm.

To more specifically determine how much of the MARK3 and MARK4 staining was found in GVDs we performed double-immunofluorescence staining with MARK3 or MARK4 and CK1δ, respectively. We then scanned several GVD-containing neurons/case where both markers were present with confocal microscopy and quantified the number and size of intracellular particles that were single stained (MARK3^+^, MARK4^+^ or CK1δ^+^) or double-stained (MARK3^+^CK1δ^+^ or MARK4^+^CK1δ^+^). Although intracellular MARK3 granules almost invariably coincided with the presence of CK1δ granules, only 10% of the granules were MARK3^+^CK1δ^+^. The remaining granules consisted of 47% MARK3^+^ and 43% CK1δ^+^ (Figure 4A). We found that MARK4^+^ granules accounted for about 42% of the total number of intracellular granules, 26% were CK1δ^+^ and 32% were MARK4^+^CK1δ^+^ (Figure 4B).

**Figure 5:**
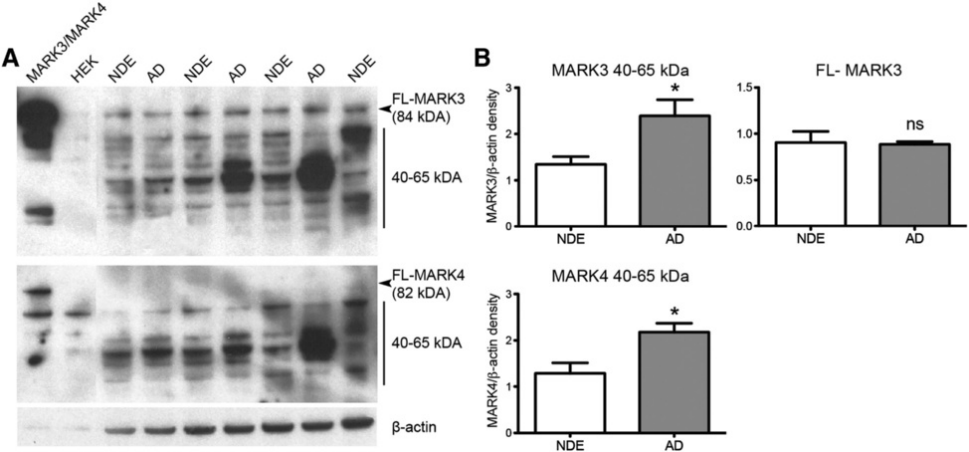
**Western blot of MARK3 and MARK4 in hippocampal homogenates. (A)** Western blots showing MARK3 (top) and MARK4 (bottom) immunoreactivities in hippocampal homogenates from 4 NDE and 3 AD cases. HEK cells transfected with either MARK3 or MARK4, respectively, served as positive controls, whereas non-transfected HEK cells served as negative control. β-actin served as a normalizing control for protein content in the homogenates. **(B)** Quantification of full-length (84 respective 82 kDa) and shorter (40–65 kDA) band densities normalized against β-actin band density. No full-length MARK4 band could be identified or measured. Data depicts means ± SEM. *p < 0.05, ns non-significant by Student’s unpaired two-tailed t-test.

To further quantify the expression of MARK3 and MARK4 we performed Western blotting of hippocampal homogenates from NDE and AD cases. Preparations of HEK cells transfected with MARK3 or MARK4 were used as positive control, and here the MARK3 and MARK4 antibodies recognized bands that corresponded to the full-length isoforms at 84 and 82 kDA, respectively. In hippocampal homogenates full-length MARK3 could be detected and measured (Figure 5A), but there was no significant difference between NDE and AD cases when the band density was normalized against β-actin (Figure 5B). Remarkably, no full-length MARK4 was detectable in the brain samples (Figure 5A). This was not due to a problem with the antibody, since a full-length MARK4 band was clearly detectable in MARK4-transfected cells. However, both antibodies recognized a number of bands in the 40–65 kDa region, and when the densities were normalized against β-actin a significant increase in AD over NDE cases was observed on both MARK3 and MARK4 blots (Figure 5B).

### MARK4 co-expresses with p-tau Ser^262^ in a Braak-stage dependent manner

**Figure 6:**
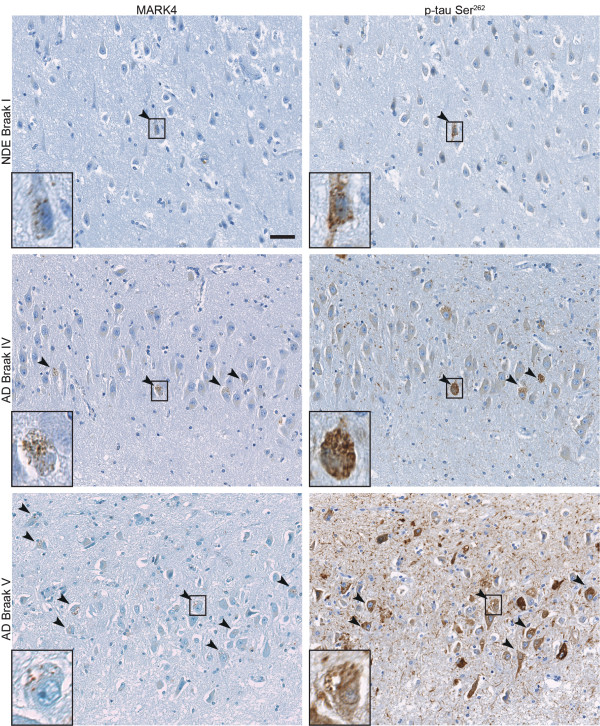
**Expression of MARK4 and p-tau Ser** ^**262**^ **in hippocampi with increasing neurofibrillary pathology.** Photomicrographs of exactly adjacent sections from the CA1-CA2 field of three cases (#6, #31 and #29) with increasing degree of neurofibrillary pathology, stained with MARK4 and p-tau Ser^262^ antibodies. Arrowheads indicate neurons that stain positive for MARK4, which increase with Braak stage. Those neurons in the adjacent section are also intensely stained with the p-tau Ser^262^ antibody. Scale bar: 50 μm.

**Figure 7:**
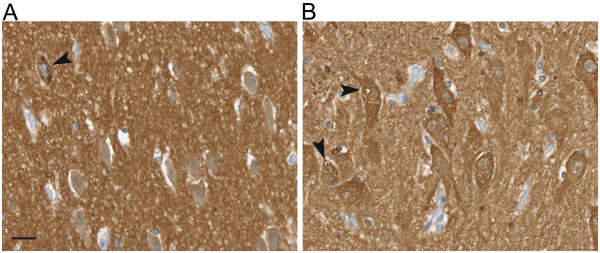
**Expression of total tau in the hippocampus.** High magnification micrographs of one NDE (**A**; #6) and one AD (**B**; #34) case stained with an antibody towards non-phosphorylated tau (tau-13). **(A)** Tau-13 immunoreactivity in the NDE case was strong in the neuropil and weak in the soma, except in NFT-containing neurons (arrowhead). However, granular immunoreactivity or GVD staining was not observed with the tau-13 antibody. The AD case **(B)** contained overall more intense staining in the somatodendritic compartment (arrowheads), but tau-13 immunoreactivity was not enriched in GVDs. Scale bar: 25 μm.

Since MARK4 was the major isoform present in GVDs and most strongly elevated in AD cases, we chose to continue to study the MARK4 isoform in relation to neurofibrillary changes. We stained adjacent sections of eight cases with the MARK4 antibody and an antibody specific for the phosphorylated Ser^262^ site of tau. We observed an increase in the number of MARK4 positive neurons with increasing Braak stage. Interestingly, this seemed to correlate very well with tau phosphorylation at the Ser^262^ epitope, which is a MARK substrate. Practically every neuron that contained MARK4 immunoreactivity also contained phosphorylated Ser^262^ tau. Remarkably, this held true even for the earliest Braak stages, whereby the few neurons containing MARK4 also had phosphorylated Ser^262^ tau (Figure 6). Tau itself was not enriched in the GVDs (Figure 7).

### MARK4 in GVDs is in a phosphorylated and activated form

**Figure 8:**
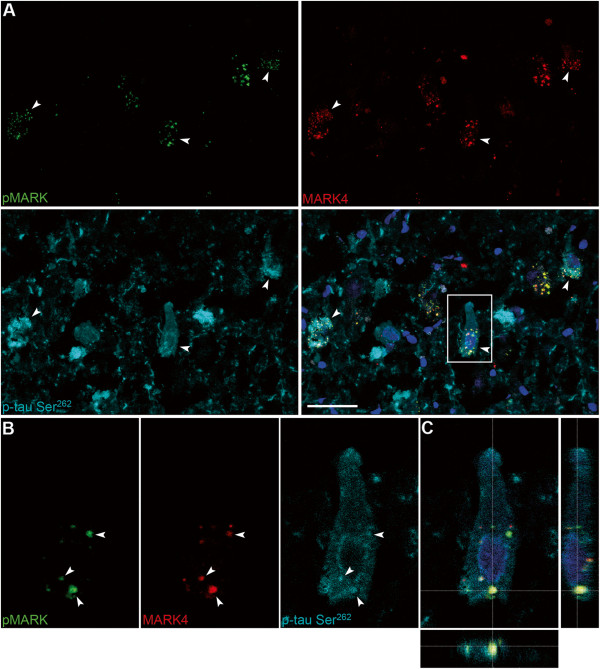
**Colocalization of MARK4, phospho-MARK, and p-tau Ser** ^**262**^ **in GVDs.** Triple-immunofluorescence images from the CA1-field of an AD case (#32) with antibodies against pMARK (FITC), MARK4 (Cy3) and p-tau Ser^262^ (Cy5). **(A)** Arrowheads indicate neurons that stain positively for all three antibodies. **(B)** A typical pre-tangle neuron with perinuclear phospho-tau staining is magnified, where arrowheads indicate individual GVDs where all three markers colocalize. **(C)** A 3-D reconstruction of a Z-stack focused on the GVD in the bottom part of the neuron. Scale bar: 100 μm.

Since MARKs can be activated by phosphorylation in the activation loop [33] we wanted to determine if MARK4 was phosphorylated in GVDs. We therefore used an antibody that recognizes the phosphorylation of threonine in the activation epitope common for all MARK isoforms (Thr^214^ on MARK4). We determined that the phospho-specific MARK antibody produced a staining pattern very similar to that of the MARK4 antibody, which was confirmed by double-immunofluorescence. This indicates that MARK4 is phosphorylated and present in an activated form in GVD neurons. When sections were triple-labeled with MARK4, pMARK, and p-tau Ser^262^ antibodies, the phosphorylated MARK4 protein often coincided with p-tau Ser^262^ staining (Figure 8).

**Figure 9:**
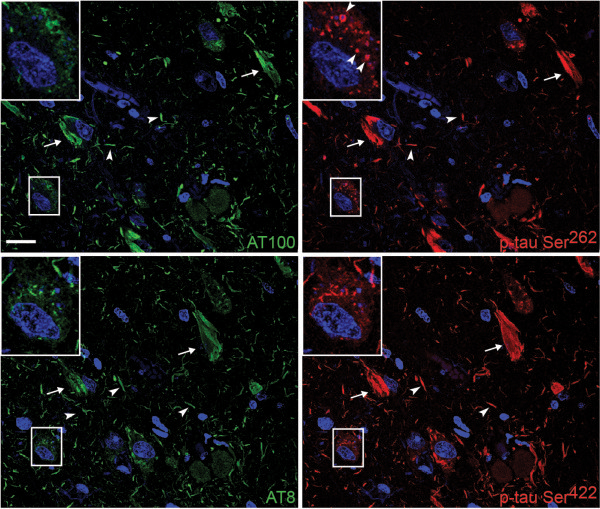
**Examination of tau phosphorylation sites in GVDs.** Double immunofluorescence of two adjacent sections from the CA1 of an AD case (#18) with four p-tau specific antibodies: Ser^212^/Thr^214^ (AT100, FITC), Ser^262^ (Cy3), Ser^202^/Thr^205^ (AT8, FITC), Ser^422^ (Cy3). Neurofibrillary tangles (arrows) and neuropil threads (arrowheads) are stained with all four antibodies. The magnified neuron is stained with all four antibodies, and only the Ser^262^ site can be observed in granular deposits in the cytoplasm (arrowheads in inset), corresponding to GVDs. Intraneuronal accumulation of fibrillar tau is stained with all antibodies. Scale bar: 25 μm.

To investigate whether other non-MARK p-tau epitopes were present in GVDs we stained sections with the p-tau Ser^262^ antibody as well as three well-characterized antibodies towards p-tau Ser^202^/Ser^205^ (AT8), Ser^212^/Thr^214^ (AT100) and Ser^422^. Interestingly, while all four antibodies stained fibrillar tau and neuropil threads, only the p-tau Ser^262^ antibody stained granular deposits in the soma (Figure 9).

## Discussion

The mRNA levels of all MARKs were similar between AD and NDE and did not correlate with pathological changes. This indicates that transcription of MARK genes is unaffected in AD. Whereas the uniform neuronal expression of MARK1, 2, and 3 mRNAs was reflected by a cytoplasmic expression of the corresponding protein in neurons, there was no noticeable cytoplasmic MARK4 protein expression and hence MARK4 was entirely absent in healthy neurons. This absence was not due to lack of MARK4 affinity, as the antibody detected MARK4 in co-transfected HEK cells. In a previous study we observed low cytoplasmic MARK4 expression in NDE cases using the *in situ* proximity ligation assay [13]. This is likely due to the higher sensitivity achieved using the proximity ligation method, but could also reflect the recognition of neurons in which pathological MARK phosphorylation has begun. It is worth noting that both fresh-frozen and formalin-fixed paraffin embedded tissues were used, with identical staining pattern. The very low MARK4 protein expression in NDE cases was confirmed by Western blotting, which did not detect full-length MARK4. This apparent absence of MARK4 protein may be due to that the expression level is below the limit of detection using the present methodologies, but could also perhaps be due to some unknown post-translational modification at the epitope site that disturbs both immunohistochemical and biochemical detection in the post-mortem tissues.

In contrast to the very low expression in NDE, we observed a prominent expression of MARK4 protein in AD tissues. It is interesting that MARK4 is specifically elevated, particularly since a locus near the MARK4 gene has recently been described to have genome-wide significance for AD [34]. MARK3 and MARK4 granules coincided with the presence GVDs (as determined by CK1δ^+^ granules), which are defined as electron-dense granules within double membrane-bound cytoplasmic vacuoles, and that are believed to be part of the autophagic system [22, 32]. Interestingly, it was demonstrated in a recent report that the level of GVDs increases steadily with AD progression, correlating with Braak stage, CERAD score, amyloid beta phase and cognitive state, but does not correlate in other non-AD tauopathies [30]. Another recent study corroborates the view that neurofibrillary and GVD pathologies are tightly associated [35].

The distribution of MARK3 and MARK4 granules in CA-neurons correlated well with CK1δ granules and all three markers increased in a Braak-stage dependent manner, except for a small attenuation in CA1-CA2 at Braak stage VI. This attenuation is likely due to the severe neuron loss observed in this area at this Braak stage, since the data was expressed in neurons/mm^2^ and not percent of total neurons. Interestingly, only 32% of intracellular granules were MARK4^+^CK1δ^+^ and as few as 10% were MARK3^+^CK1δ^+^. Furthermore, MARK4^+^ granules were significantly larger in size than CK1δ^+^ or MARK4^+^CK1δ^+^ granules. Taken together, this suggests that although MARK3 and MARK4, along with CK1δ are sequestered in a GVD-dependent manner, CA-neurons utilize different intracellular pathways to sequester these kinases. The relatively large size of the MARK4^+^ granules as compared to the CK1δ^+^ granules could possibly indicate that MARK4^+^ granules represent a more mature form of GVDs.

The exact role of these events in neuropathological progression in AD remains to be elucidated. It is well established that there is a spatiotemporal progression of neurofibrillary pathology in the brain, with certain areas being affected before others. The entorhinal cortex becomes affected at an early stage with subsequent appearance of NFTs in the CA1-field [36]. Conversion of individual neurons into NFTs is also suggested to occur in a hierarchical sequence of phosphorylation events, with Ser^262^ phosphorylation appearing early in the formation of a NFT when the neuron is in a pretangle stage [9, 37]. Herein we demonstrate that the first appearance of Ser^262^ phosphorylation in the CA-field is paralleled by an activation of the GVD system and particularly by strong MARK4 expression. In CA1 in the early Braak stages, tau Ser^262^ is initially only evident in a very limited number of neurons, and although present in the cytoplasm is highly concentrated in the GVDs. According to the GVD-staging proposed by Thal *et al*. [30], GVD pathology first appears in CA1-neurons and only in later stages in the entorhinal cortex. The fact that GVD formation is first apparent in CA1-neurons could be due to a higher sensitivity of these neurons to tau phosphorylation, or alternatively a higher ability to engage autophagic GVD processes as a defence mechanism. In concordance with this latter hypothesis, induction of autophagy has recently been shown to be able to reduce levels of phosphorylated tau in neurons [38]. It can be hypothesized that this early phosphorylation of tau Ser^262^ is driven by increased activities of cytoplasmic MARK2, MARK3 and very low levels of MARK4. This hypothesis is partly supported by the increased association of MARK2 and MARK4 to tau in the somatodendritic compartment, shown in a previous study [13]. Phosphorylation at tau Ser^262^ will lead to subsequent phosphorylation at other tau epitopes which will cause detachment from microtubules and make tau available for aggregation into NFTs [10].

MARK4 in the GVDs was highly phosphorylated, indicative of the presence in a high activity form. MARKs contain numerous kinase-regulated sites [39, 40] and, for example, phosphorylation at the Thr^214^ site (of MARK4 sequence) can increase MARK activity >50 times [33]. However, the exact mechanism involved in activation of MARK4 in AD, and the possible link to amyloid beta oligomers [41] remains to be established.

It is possible that MARK3 and 4 are phosphorylated and activated within the GVDs, but alternatively the activation may already have occurred in the neuronal cytoplasm. If so, there appears to be a very effective sequestering by the GVDs since we could not detect any phosphorylated and activated MARK4 or MARK3 in the cytosolic compartment. MARK4 in GVDs appears to undergo proteolysis since only shorter bands were observed in the immunoblots and these bands had increased in intensity in AD. That the 40–65 kDa bands represent proteolytic products within the GVDs is also consistent with the lack of similar bands in HEK cell lysates. However, it cannot be excluded that they to some extent represent non-specific cross-reactive bands.

The detection of bands representing truncated protein evident in the NDE cases probably reflects the rare presence of GVDs at these early Braak stages. A similar pattern was observed for MARK3, whereby an increase of truncated forms of MARK3 in immunoblots corresponds to an increased expression in AD by appearing in a minor fraction of GVDs. In contrast, full- length MARK3 was not elevated in AD and therefore presumably reflects the cytoplasmic component that is similar between NDE and AD cases.

It is possible that MARK4 and to some extent MARK3 become sequestered and that they phosphorylate tau already present in the GVDs at Ser^262^, due to the closer proximity between substrate and enzyme [13], as well as due to the activated state of MARK3 and MARK4. Alternatively, Ser^262^ may already be phosphorylated in the tau molecules sequestered into the GVDs, and sequestering of tau phosphorylated Ser^262^ into GVDs would confer prevention of subsequent phosphorylation at other epitopes and tangle formation. In fact, subsequent phosphorylation at Ser^202^/Thr^205^ (AT8), Thr^212^/Ser^214^ (AT100), and Ser^422^ are absent in the GVDs, in spite of the presence of many tau phosphorylating enzymes such as CK1δ [26, 28], GSK3β in its active form [27] and CDK5 [42]. It should be noted that our results on this matter conflict slightly with those of Leroy *et al.*[27] who found not only p-tau Ser^262^ but also AT100 immunoreactivity (although to a lesser extent) in GVDs. An early study also identified GVD immunoreactivity using the Tau-1 antibody in dephosphorylated brain tissues, suggesting the presence of the p-Ser^199^ epitope [1, 43]. In further support of this hypothesis, Ikegami *et al.* could not find PHF-structures upon ultrastructural examination of GVDs, indicating the presence of pre-PHF tau [44]. It should be noted though that total tau does not accumulate into GVD as a result of such possible sequestering. Moreover, it is interesting to note that CaMKII, which accounts for a large portion of normal brain p-Ser^262^ activity [45] has not been found in GVDs in three immunohistochemical studies of AD hippocampus [46–48].

The cytoplasmic phosphorylation of tau steadily increases with time, likely driven by cytosolic MARK2. This notion is supported by an increased MARK2-tau interaction in this compartment [13, 21], as well as a complete lack of immunoreactivity in GVDs. Hence a continuous cytoplasmic presence of active MARK2 may allow a slow but steady continuous cytosolic phosphorylation at other p-tau sites, a process that GVD-mediated autophagy cannot prevent. The presumed protective effect of GVDs ultimately fails at later Braak stages, either due to a diminished capacity of GVDs to sequester p-tau Ser^262^ or due to breakdown of GVDs with subsequent release of their components into the cytosol. Together these events lead to the cytoplasmic neuronal compartment filling up with tau phosphorylated at multiple sites available for further aggregation into NFTs, which in turn may result in neuronal degeneration and associated cognitive decline.

There is presently no evidence for an involvement of MARK1 in any of these pathological events. A previous investigation of MARK expression in human brain tissues reported increased levels of MARK1 in AD in tau tangle-containing neurons [20], in contrast with current and previous [13] findings, and this discrepancy could be due to less specific antibodies used in that study. Another report described an association between active MARK and neurofibrillary tangles but did not further describe the antibody characteristics [49].

## Conclusion

In the present study we demonstrate that phosphorylated MARK4 and to some extent MARK3 are highly localized to GVD expressing neurons and that this expression is strongly correlated with phosphorylation of tau at Ser^262^. This may represent a cellular defense mechanism to remove activated MARK and p-tau Ser^262^ from the cytosol, thereby reducing the phosphorylating effect on tau Ser^262^ that appears to be a critical step for subsequent neurodegeneration in AD.

## Competing interest

The authors declare that they have no competing interest.
